# Monte-Carlo value analysis of High-Throughput Satellites: Value levers, tradeoffs, and implications for operators and investors

**DOI:** 10.1371/journal.pone.0222133

**Published:** 2019-09-11

**Authors:** Fan Geng, David B. Gomez, Yue Guan, Joseph Homer Saleh

**Affiliations:** School of Aerospace Engineering, Georgia Institute of Technology, Atlanta, GA, United States of America; Shandong University of Science and Technology, CHINA

## Abstract

High-Throughput Satellites (HTS) are a distinctive class of communication satellites that provide significantly more throughput per allocated bandwidth than traditional wide-beam communication satellites. They are the proverbial wave of creative disruption in the space industry and are poised to disrupt the communication market in significant ways. The objective of this work is to develop a decision-analytic framework for assessing the value of High-Throughput Satellites and to provide meaningful results of the value of such systems under realistic design, operational, and market conditions. We develop the cost and revenue models of HTS. To build the revenue model, we develop a hybrid data-driven and scenario-based load factor model that combines historical data based on financial records from current HTS operators with extrapolations based on best-, nominal-, and worst-case scenarios. We then integrate the cost and revenue models within a stochastic simulation environment and perform Monte-Carlo analysis of the *net present value* (*NPV*) of HTS. One important result is that a medium-sized HTS significantly outperforms a roughly equivalent traditional wide-beam satellite, even under the worst-case loading scenario. Another important result, here identified and quantified, is the tradeoff between the *average revenue per user* (*ARPU*) and average loading of the satellite and how it is mediated by the downlink speed provided to consumers. This result can be used in different ways, for example, by helping define the boundaries of what is competitively achievable in terms of *ARPU* and downlink speed offerings. The implications of these results are that they delineate the pathways to financial failure and the boundaries beyond which an HTS will be value-negative, or alternatively, the asymptotic minimum values for an HTS to be value-positive.

## 1. Introduction

On October 4, 1957, a small beeping satellite, Sputnik, heralded the beginning of the Space Age. From this humble start, the space industry grew into a multi-hundred-billion-dollar industry five decades later [1]. This is a small-volume industry, with fewer than 10,000 satellites launched to date, and less than 20% of these constitute the current active fleet that underpins this industry. Spacecraft today fulfill a myriad of functions, from Defense and Intelligence (e.g., early warning, reconnaissance), to Science (e.g., Earth observation, interplanetary probes), to Navigation and Communication functions.

In April 2019, two announcements were met with a collective shrug by the general press, and tepid enthusiasm from the aerospace press, that Amazon is planning a High-Throughput Satellite (HTS) constellation for broadband access with over 3,000 satellites in Low Earth Orbit (project Kuiper), and that SpaceX obtained FCC approval to lower the orbit of nearly 1,600 satellites in its planned HTS constellation (project Starlink).

These announcements, we propose, are the tip of the iceberg of another Sputnik-like milestone in the Space Age. While much attention in the media and public discourse on space issues has focused on launch vehicles and their reusability, the more important but quieter revolution is unfolding in satellite communication with HTS, and it will usher a new era of broadband connectivity in the next decade. High-Throughput Satellites, we noted in a companion article [2], are the proverbial wave of creative disruption in the space industry. Creative disruption refers to the process of major technological innovation by which new products displace older ones or render them obsolete, and in so doing, they disrupt economic structures and market conditions and create new ones. High-Throughput Satellites will reshape the space and telecommunication industry in significant and meaningful ways in the next decade. They will affect broadband access worldwide including connecting the unconnected, will create new business models, and will likely spur new applications for ubiquitous communications.

**Fig 1:**
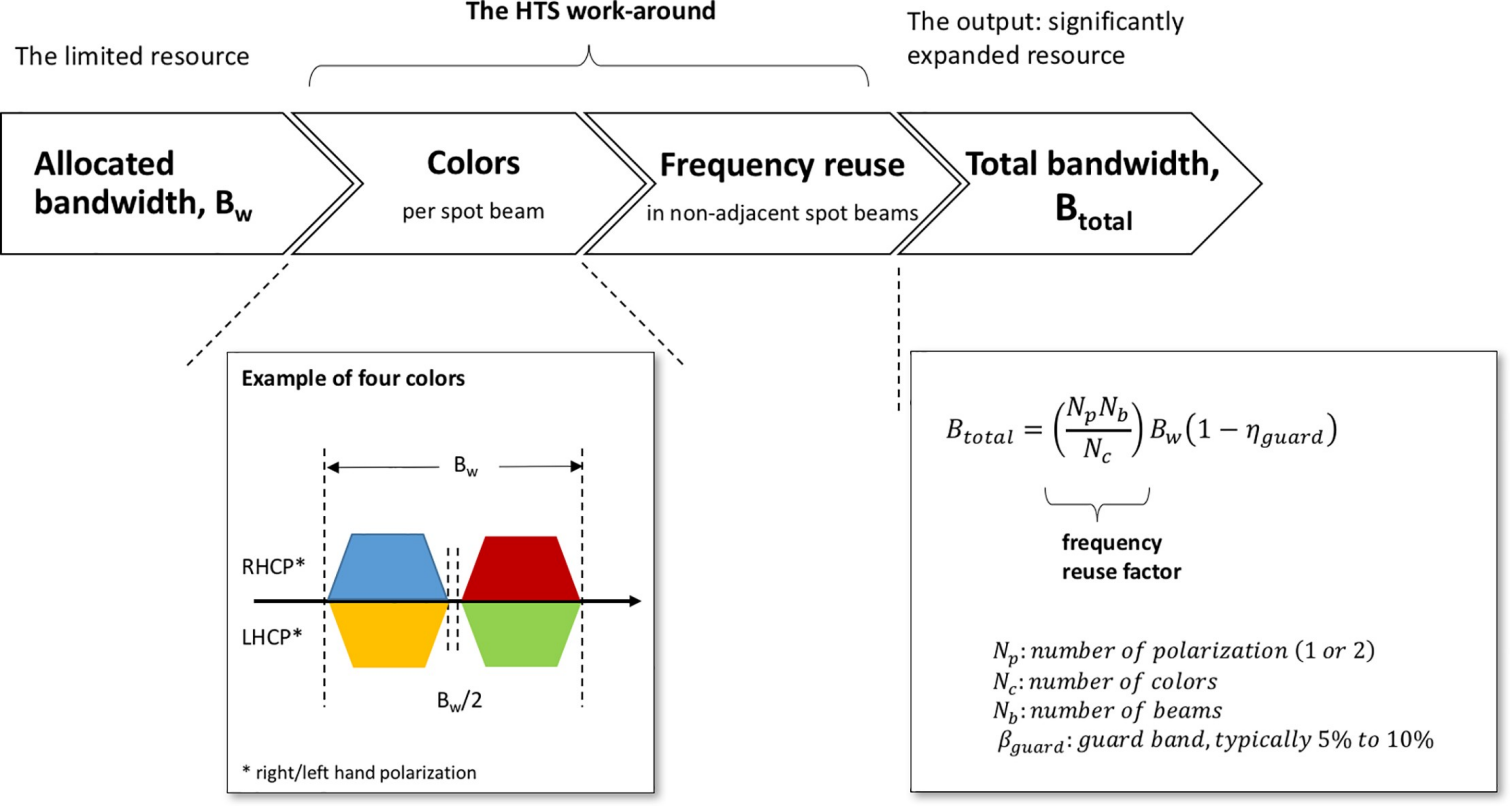
Defining features of hight-throughput satellites.

What are High-Throughput Satellites? And why this assessment? We examined these issues in a companion article entitled, “Review of High-Throughput Satellites: market disruptions, affordability-throughput map, and the cost per bit/second decision tree,” [2]. Very briefly, HTS is a distinctive class of communication satellites. They are defined and enabled by two related technological features: (1) the use of spot beams or narrow beams covering a small geographic area, with a large number of beams tessellated together like a mosaic to cover a region of interest; and (2) the frequency reuse of the allocated bandwidth to the satellite in non-adjacent spot beams. Together, these features result in significantly higher bandwidths, and consequently higher throughputs, than traditional wide-beam satellites (see Fig 1).

In addition to this massive increase in bandwidth, the affordability of the bits per second or cost of throughput with HTS is significantly lower than that with conventional wide-beam satellites—by one or two orders of magnitude—and it is reaching terrestrial-like economics [2]. These bundled features of HTS, considerable throughput, ubiquitous coverage, and affordable (broadband) connectivity are the main drivers of the disruptions that will be brought by these satellites.

Two milestones in the timeline of advent of HTS are worth noting: the first HTS launch with Thaicom 4 in 2005 with 45 Gbps, and the launch of ViaSat 2 in 2017 with over 300 Gbps. Over this time period 2005–2017, the yearly launch rate of HTS has increased tenfold. A single HTS can now provide as much throughput as the entire fleet of traditional (wide-beam) communication satellites in GEO. The advent of HTS and the onset of obsolescence of traditional wide-beam communication satellites are driven jointly by cost considerations and by the changing nature of communication markets, in particular how people and businesses access and consume data [2]. More specifically, the mix in demand for connectivity has shifted dramatically from point-to-multipoint broadcast communications to point-to-point broadband connectivity. These new market demands for ubiquitous broadband connectivity are ill-served by wide-beam satellites, which are optimized for broadcast efficiency but do not fare well when used outside their shrinking and niche markets.

We examined in the companion article both the technical details of HTS and the disruptions they are likely to bring to the space industry, in particular how manufacturers and operators will be impacted by these systems. The reader interested in this discussion is referred to [2]. As the list of companies planning HTS constellations for broadband connectivity continues to grow [3–6], the following questions will become ever more pressing:

- How valuable are these satellites, and as a co-requisite, how can their value be assessed?
- Under what conditions, given technical and operational (e.g., design and service pricing), as well as market uncertainty, will an HTS system be financially successful? What are the pathways to success or failure?

These are some of the issues we examine in this article. More broadly, the objective of this work is both to develop a decision-analytic framework for assessing the value of High-Throughput Satellites and to provide meaningful results of the value of such systems under realistic design, operational, and market conditions. Ultimately, these analytics and results are meant to help inform design, cost, and service pricing considerations, among other things, for these systems to be successful.

The theoretical underpinning of this work is the notion that an engineering system is a value-delivery artefact; its value derives from the flow of service it provides to different stakeholders. This is a central idea for the design and acquisition of space systems, and it is the conceptual pillar upon which this work rests. Given this underpinning, and since there are markets and quasi-rent for the services provided by the communication satellites here examined, discounted cash flow techniques are the standard method for capturing the value of such systems. This is the reason for the adoption of *net present value* (*NPV*) as well as pseudo *Return on Invested Capital* (*ROIC*) calculations in this work. Furthermore, it is well-established that decision-makers do not just make decisions based on expected values alone but account for some measure of uncertainty as well in their deliberations. Monte-Carlo analysis allows us to conveniently propagate the intrinsic uncertainty in the analysis, and it provides the readers and decision-makers with more transparent and useful results than simply expected values of *NPV* and *ROIC*.

The remainder of this article is organized as follows. In section 2, we provide a brief review of the previously developed value model of traditional wide-beam communication satellites. This will serve as a basis for the remainder of this work and against which the HTS value model will be benchmarked and contrasted. In section 3, we develop the cost, revenue, and value models for HTS. In section 4, we conduct Monte-Carlo value analyses under different market and operating scenarios, and we provide a comparative analysis of HTS with wide-beam satellites. We discuss our findings and conclude our work in section 5.

## 2. Traditional wide-beam communication satellite: Review of value model

In this section, we provide a brief review of the cost, revenue, and *net present value* (*NPV*) model of a traditional wide-beam communication satellite developed in [7, 8]. We conclude with an application of said value model and a brief discussion of its results. This section serves as a background and foundation on which we build and later contrast the value model of HTS.

### 2.1. Cost model of traditional wide-beam communication satellites

There are different levels of resolution for examining the cost structure of a satellite and aggregating its life cycle cost (LCC). One commonly used distinction in the space industry is the breakdown of LCC into cost to initial operational capability (IOC), which includes all costs incurred up to the delivery of the satellite on-orbit, and cost of operations incurred afterward. The model for the cost to IOC is given by Eq (1).

$$C_{IOC}=(C_{acq.}+C_{launch})\cdot (1+IR)$$ (1)

$$\left\{ \begin{array}{ll} C_{IOC} & :cost\;to\;IOC\\ C_{acq.} & :acquisition\;cost\\ C_{launch} & :launch\;cost\\ IR & :insurance\;rate \end{array}\right.$$

For this brief review, it is sufficient to model the acquisition cost of a communication (wide-beam) satellite as a function of the size of its payload, more specifically, as a function of the number of 36-MHz equivalent transponders carried onboard, *N*, as given in Eq (2). More involved satellite acquisition cost models would account for other factors such as mass, power, and propulsion system type on board the satellite. These additional details are not relevant for our present purpose.

The model in Eq (2) is adapted from [8] for a wide-beam communication satellite with a design life of 15 years. Along with the size of the payload, the model also accounts for the marginal cost of durability, *δ*, of the spacecraft. This reflects the incremental cost incurred to the spacecraft for one additional year of design lifetime [9].

$$C_{acq.}=63.1\cdot \ln\left(N\right)-166.3\cdot {\left(1+\delta \right)}^{15-T_{service}}\;\$\;million$$ (2)

$$\left\{ \begin{array}{ll} N & :number\;of\;36\;MHz\;equivalent\;transponders\\ \delta & :marginal\;cost\;of\;durability\\ T_{service} & :service\;lifetime\;of\;satellite\\ R^{2} & =0.89\\ for & 24\leq N\leq 92 \end{array}\right.$$

The second category of costs refers to the ongoing cost of operation, *C*_*ops*_, of the satellite over the duration of its life on-orbit, *T*_*service*_. The present value of the satellite’s cost of operation, *PV(C*_*ops*_*)*, is given by Eq (3), and it accounts for the discount rate, *r*, the growth/decline rates of the initial costs of operation, γ, and the launch delays, *ΔT*, if any.

$$PV(C_{ops})=\sum_{i=1}^{T_{service}}\frac{C_{ops,0}{\left(1+\gamma \right)}^{i-1}}{{\left(1+r\right)}^{i+\Delta T}}$$ (3)

When aggregated, the cost to IOC and present value of the cost of operation determine the present value of the life cycle cost, *PV(LCC)*, of the satellite. This is given by Eq (4) and constitutes the lifecycle cost model of traditional wide-beam communication satellites.

$$PV(LCC)=C_{IOC}+PV(C_{ops})$$ (4)

We develop the revenue model next, following which we integrate both the discounted cost and revenue models to produce a *net present value* model of traditional wide-beam satellites.

### 2.2. Revenue model of traditional wide-beam communication satellites

The revenue model of the satellite is more involved than its cost model, with several additional parameters or degrees of freedom to capture different services and revenue streams. Some parameters are common between the cost model and the revenue model, such as the time delay between when the acquisition cost is incurred and when the costs of operation begin, *ΔT*, as well as the discount rate, *r*. The load factor is a necessary component of the revenue model; it is defined as the percentage of transponders onboard the satellite that are leased, and it is subject to change over time. The first expression for the load factor takes the form given by Eq (5).

$$L(t)=\left\{ \begin{array}{ll} 0 & for\;t\leq \Delta T\\ L_{0}\left[1-\exp\left(-\frac{t-\Delta T}{\tau }\right)\right]+N(0,\sigma^{2}) & for\;t>\Delta T \end{array}\right.$$ (5)

$$\left\{ \begin{array}{ll} L(t) & :load\;factor\\ L_{0} & :steady-state\;load\;factor\\ \tau & :dynamic\;time\;constant \end{array}\right.$$

$N(0,\sigma^{2})$ is a noise factor with a small variance to account for some stochasticity in the loading dynamics but keeps it confined to the 0–100% range. The time constant τ reflects the time needed to reach 95% of the steady-state load factor *L*_*0*_, e.g., 3*τ* = *t*_95%_.

The load factor can be broken down into various services for which the transponders are leased, such as audio, data, and video, which can have different lease prices in different markets and for different lease durations. The average revenue generated by service *i*, ${\bar{u}}_{i}(t)$, is given by the product of three terms: the first is the number of leased transponders, *L*(*t*)∙*N*; the second is the average service mix, ${\bar{s}}_{i}$; and the third is the average lease price of service *i*, ${\bar{P}}_{i}$. The average revenue per service, ${\bar{u}}_{i}$, is given by Eq (6), and the total revenue generated per year, ${\bar{u}}_{total}(t)$, is given by Eq (7).

$${\bar{u}}_{i}(t)=[N\cdot L(t)]\cdot {\bar{s}}_{i}(t)\cdot {\bar{P}}_{i}(t)$$ (6)

$${\bar{u}}_{total}(t)=\sum_{i}{\bar{u}}_{i}(t)$$ (7)

**Fig 2:**
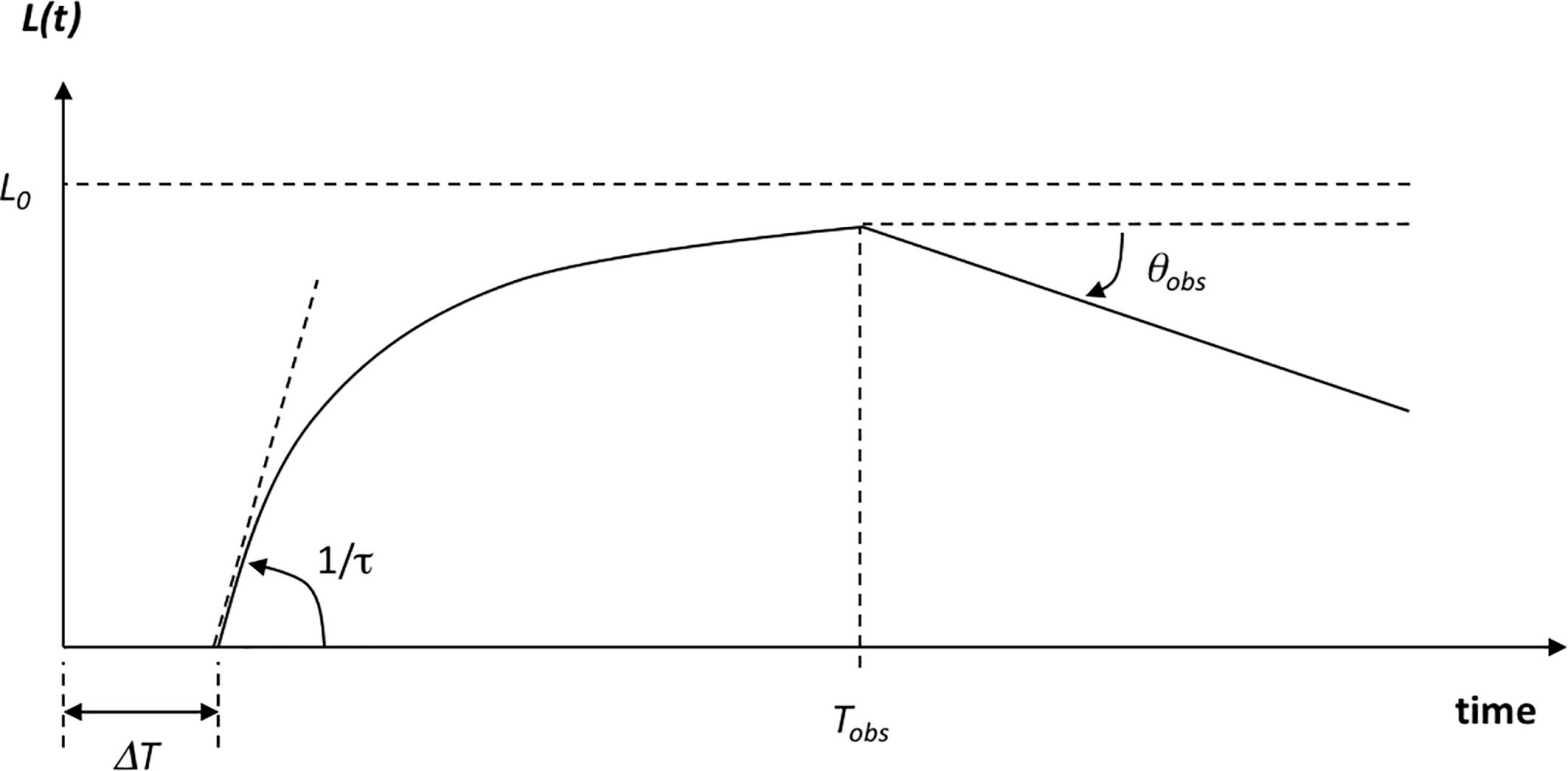
Parameters of our (wide-beam) satellite loading model.

Two additional degrees of freedom are included in the model that modify the expression of the load factor for more realistic market conditions, and consequently, they modify the revenue model as well. They also add to the stochastic nature of the loading model by introducing two new random variables: the first is the time to onset of obsolescence, *T*_*obs*_. This refers to the onset of customer churn as newer and better transponders are available on other satellites (covering the same geographic region) and customers begin to switch to these newer technologies. The second is the intensity of obsolescence, *θ*_*obs*_, which captures the intensity (rate) of customer churn. In the current model, a simple linear decay determines the percentage points lost per year in the satellite load factor after the time of onset of obsolescence has elapsed. There are more involved models of obsolescence available, as discussed in [2, 10, 11], but for the purposes of this work, their effects are of little relevance for small *θ*_*obs*_. The previous expression for the satellite load factor is therefore extended to *t*≥*T*_*obs*_ and is given by Eq (8). A depiction of the loading parameters is provided in Fig 2.

$$L(t)=\left\{ \begin{array}{ll} 0 & for\;t\leq \Delta T\\ L_{0}\left[1-\exp\left(-\frac{T_{obs}-\Delta T}{\tau }\right)\right]+N(0,\sigma^{2}) & for\;\Delta T<t<T_{obs}\\ L_{0}\left[1-\exp\left(-\frac{T_{obs}-\Delta T}{\tau }\right)\right]-\theta_{obs}\cdot (t-T_{obs})+N(0,\sigma^{2}) & for\;T_{obs}\leq t<T_{life} \end{array}\right.$$ (8)

The present value of the revenue, *PV(rev)*, generated by all leased transponders across different services over the life of the satellite is therefore given by Eq (9).

$$PV(rev)=\sum_{i=1}^{T_{service}}\frac{{\bar{u}}_{total}(t)}{{\left(1+r\right)}^{i}}$$ (9)

### 2.3. NPV model and Monte-Carlo value analysis of wide-beam communication satellites

The *net present value* of the satellite can now be determined by integrating the cost and revenue models discussed in the previous two subsections. This value model integrates intrinsic spacecraft design and cost considerations, with exogenous market conditions and operational choices (including service mix pricing). The *NPV* is determined as the present value of all the satellite’s revenue streams minus the present value of the life cycle costs incurred, and it is given by Eq (10).

$$\begin{array}{c} NPV=PV(rev)-PV(LCC)\\ \Leftrightarrow \\ NPV=\sum_{i=1}^{T_{service}}\frac{{\bar{u}}_{total}(t)}{{\left(1+r\right)}^{i}}-\left[C_{IOC}+\sum_{i=1}^{T_{service}}\frac{C_{ops,0}{\left(1+\gamma \right)}^{i-1}}{{\left(1+“r\right)}^{i+\Delta T}}\right] \end{array}$$ (10)

We provide next realistic calculations of the *NPV* for a traditional wide-beam satellite. The parameters selected within the model represent a medium-sized wide-beam communication satellite with 60 transponders and a design life of 15 years. Several parameters within the model were set as random variables. These can be broken down into the random variables within the cost model and those within the revenue model. A complete description of the model parameters, the random variables included, and their distributions is provided in Table A in S1 Appendix.

**Fig 3:**
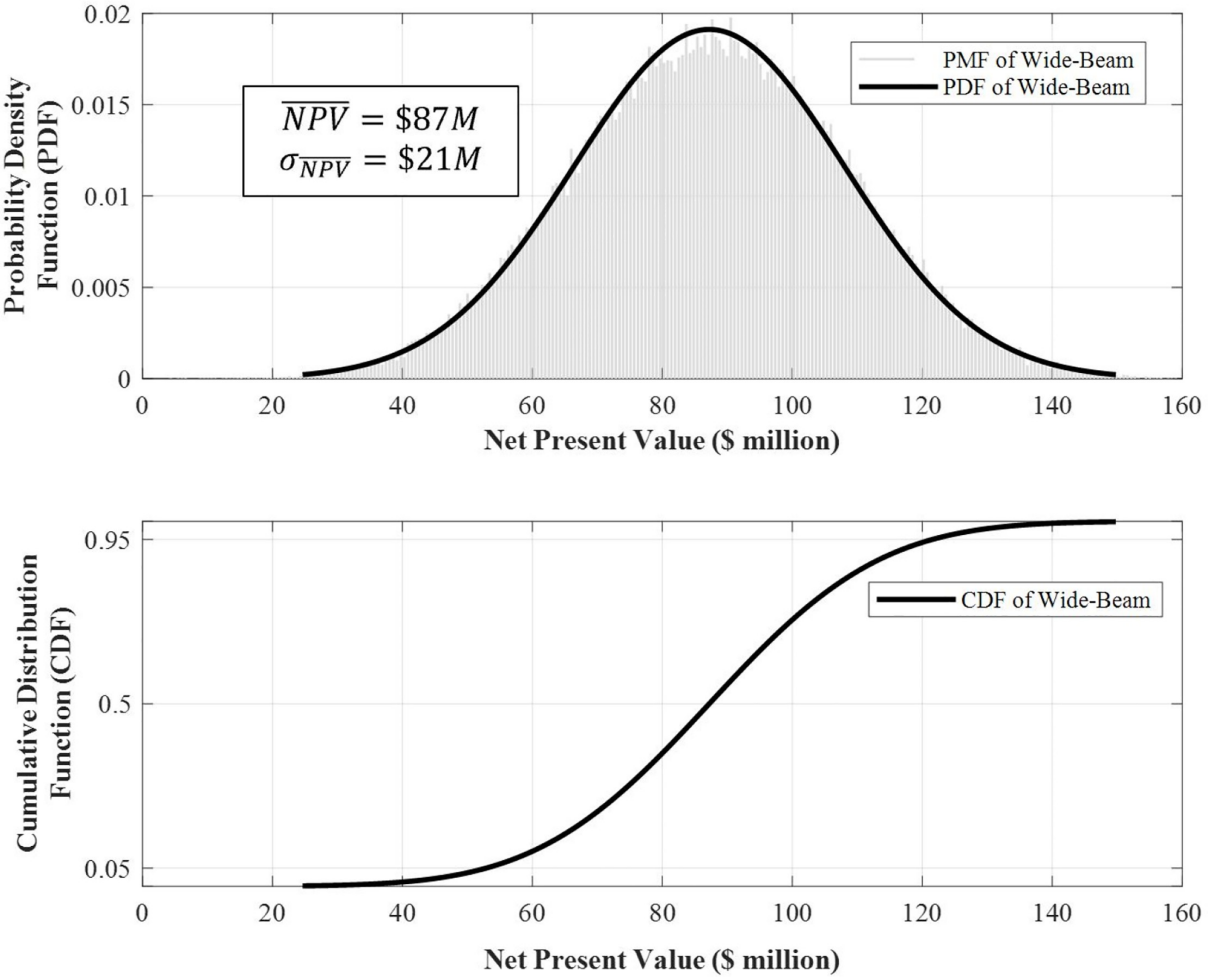
Probability density function (upper panel) and cumulative distribution function (lower panel) of the *NPV* of a 60-equivalent-transponder wide-beam communication satellite after a design life of 15 years on-orbit.

The outputs of the Monte-Carlo simulations include probability distributions of the *net present value* of the satellite after a design life of 15 years. Net value results for traditional wide-beam communication satellites are provided in Fig 3.

Fig 3 reads as follows. For our medium-sized traditional wide-beam communication satellite, the expected value of *NPV* will be $87*m* with a standard deviation of $21*m* after a design life of 15 years on-orbit. Furthermore, the 90% confidence interval spans the $53*m* to $122*m* range. The coefficient of variation, defined as the ratio of the standard deviation to the mean, is another (dimensionless) measure of volatility, and for this representative satellite, it is about 24%.

In addition to Eq (10) and the results in Fig 3, we define a *discounted Return on Invested Capital* ratio, *d–ROIC*, as follows:

$$d-ROIC\equiv \frac{Present\;Value\;of\;Revenue\;Generated}{Present\;Value\;of\;Life\;Cycle\;Costs}=\frac{PV(rev)}{PV(LCC)}.$$ (11)

The return on the invested capital ratio is traditionally defined as the ratio of the net profit to the invested capital [12]. The distinguishing feature of our *discounted-ROIC* is that it involves the present value of both the revenue and lifecycle costs, or *discounted* dollars earned per *discounted* dollar spent. Both these value metrics, *NPV* and *d-ROIC*, are random variable outputs in our Monte-Carlo analysis. Fig 3 provides the joint distribution of traditional wide-beam satellite *net present value* and discounted return on invested capital after a design life of 15 years.

**Fig 4:**
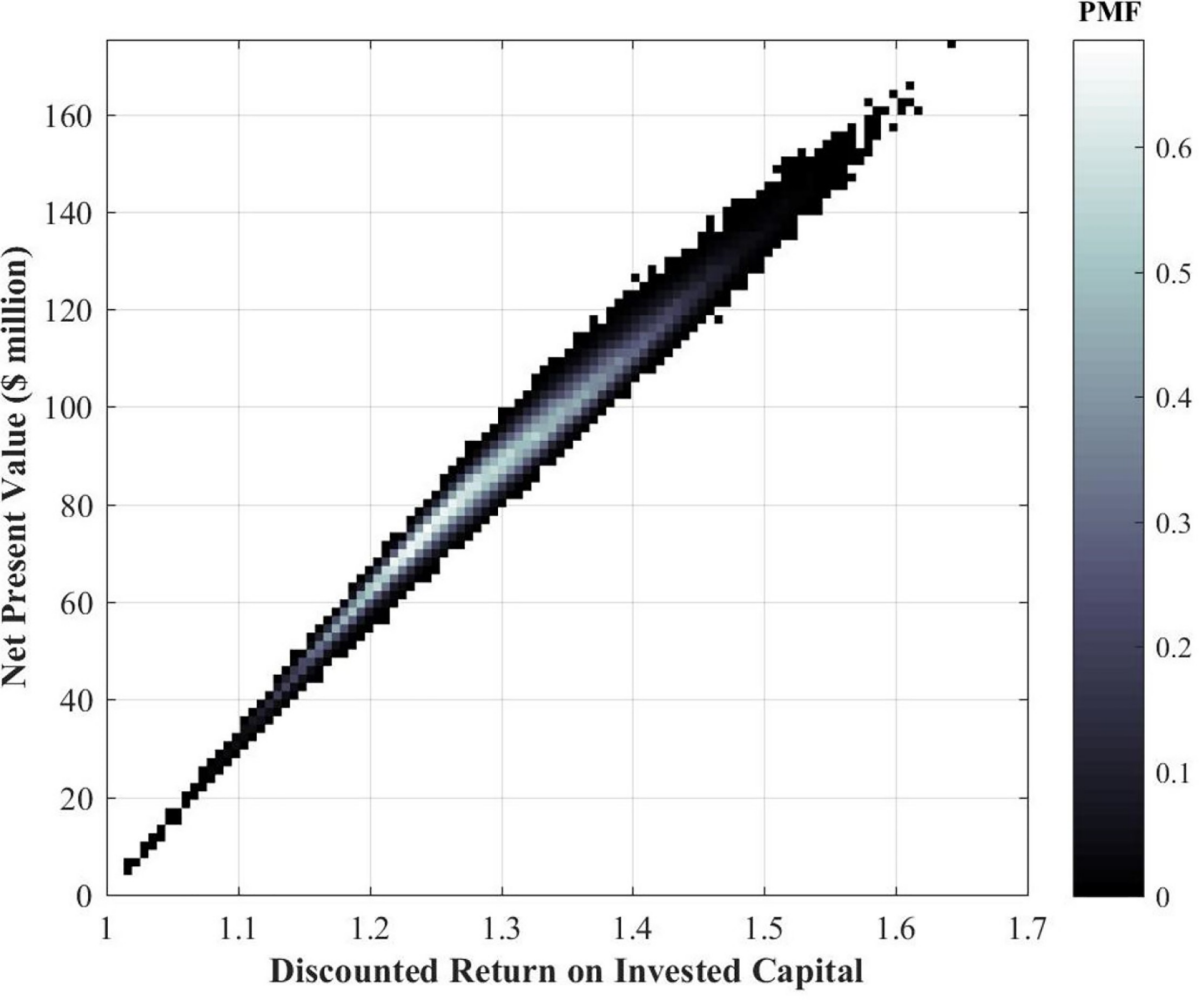
Joint distribution of traditional wide-beam satellite *NPV* and *d-ROIC* for the same 60-equivalent-transponder wide-beam communication satellite after a design life of 15 years on-orbit.

The clustering in Fig 4 indicates a strong linear relationship between the *NPV* and *d-ROIC*, with an average slope of about $2.7*m*/%. For the wide-beam satellite considered here, the expected values of *NPV* and *d-ROIC* are $87*m* and 1.3 or 30% returns, respectively, after a design life of 15 years. The dispersion in the joint distribution plot, along the x-axis for example, is simply the result of achieving the same *NPV* under different (discounted) revenues and cost profiles. Finally, it is worth noting that while such data at a single-satellite level is not available publically and is usually a closely guarded piece of information by satellite operators, the results in Fig 4 are reasonably accurate and representative of the financial performance of the class of satellite here considered, given the authors’ experience and anecdotal evidence.

## 3. High-throughput satellite: Cost, revenue, and net present value model development

In the previous section, we reviewed the development and results of the value model for traditional wide-beam communication satellites. In this section, we develop the cost, revenue, and value models of High-Throughput Satellites and discuss distinguishing features between the HTS models and those of traditional wide-beam communication satellites. The results and comparative analyses are provided in the next section.

### 3.1. Cost model of high-throughput satellites, and the affordability-throughput map

**Fig 5:**
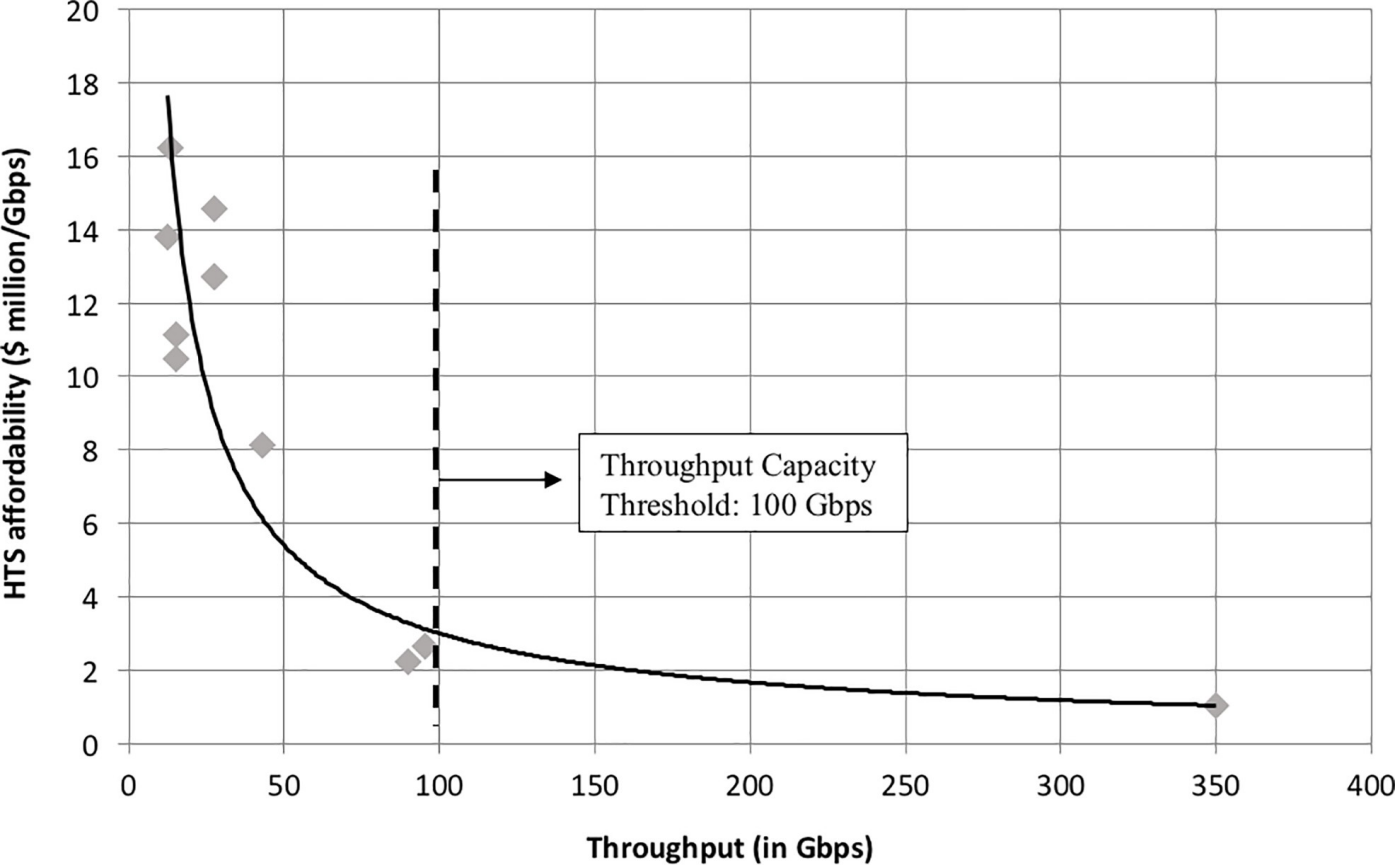
HTS affordability-throughput map with power regression model and the throughput capacity threshold.

As discussed in [2], the anchor tenant of HTS is consumer broadband. Two key metrics are essential for its broad adoption: affordability and performance. At the system level, we capture affordability with the cost per bit per second delivered by the satellite, and we capture performance by the total throughput. To develop our cost model, we examined the acquisition cost and total satellite throughput, *R*_*total*_, of HTS based on historical data [13]. The launch cost was omitted since it does not reflect any intrinsic relationship between affordability and throughput. The results for GEO HTS are shown in Fig 5 along with a least squares regression model in Eq (12).

The results in Fig 5 display three important features:

- There is a remarkable power relationship between HTS affordability and throughput, reflecting clear and substantial economies of scale in the cost of connectivity (cost per Gbps) to be reaped in designing higher throughput satellites;
- A significant amount of the variability in affordability for GEO HTS is explained by throughput alone (*R*^2^ = 0.93);
- The “knee” in the throughput-affordability curve occurs around 100 Gbps. As a result, it will become increasingly more difficult to justify the acquisition of small- or medium-sized GEO HTS below this throughput threshold of 100 Gbps.

The regression model depicted in Fig 5 is given by Eq (12) and constitutes the acquisition cost model of HTS.

$$\frac{C_{acq,HTS}}{R_{total}}=167.3{\times R_{total}}^{-0.886}$$ (12)

Along with the cost to IOC, an additional component of the cost model of HTS is the cost of operation over a given unit time period (typically a year), *C*_*ops*,*HTS*_. Compared with that of wide-beams, this cost model is more involved by necessity, as will be discussed next. The cost of operation model is given in Eq (13), and it has been tailored for two modes of operation: when there is an increase in the number of subscribers (to capture their cost of acquisition) and when there is a decrease.

$$C_{ops,HTS}(t)=\left\{ \begin{array}{ll} C_{ops,0}{\left(1+\gamma_{ops}\right)}^{t}+a\cdot ARPU\cdot \Delta N+C_{isp}{\left(1+\gamma_{isp}\right)}^{t}+b\cdot ARPU\cdot N & for\;\Delta N\geq 0\\ C_{ops,0}{\left(1+\gamma_{ops}\right)}^{t}+C_{isp}{\left(1+\gamma_{isp}\right)}^{t}+b\cdot ARPU\cdot N & for\;\Delta N<0 \end{array}\right.$$ (13)

$$\left\{ \begin{array}{ll} C_{ops,HTS} & :cost\;of\;HTS\;operation\\ C_{ops,0} & :initial\;cost\;of\;operation\\ C_{isp} & :cost\;of\;the\;internet\;service\;provider\;if\;any\\ \gamma_{ops} & :growth/decline\;rate\;of\;initial\;cost\;of\;operation\\ \gamma_{isp} & :growth/decline\;rate\;of\;cost\;of\;internet\;service\;provider\\ ARPU & :average\;revenue\;per\;user\\ a & :customer\;acquisition\;cost\;factor\;(typically\;ranges\;between\;4\;to\;8)\\ b & :internet\;service\;provider\;cost\;percentage\;(0\;to\;100\%)\\ N & :number\;of\;subscribers\\ \Delta N & :change\;in\;number\;of\;subscribers\\ t & :time\;in\;service \end{array}\right.$$

Consider the model for an increase in the number of subscribers (Δ*N*≥0). The first term represents the cost to operate the satellite, which is subject to change over time via an annual growth/decline rate for example, and it includes costs associated with facilities and employees. The second term represents the total *Customer Acquisition Cost* (*CAC*) to the operator over the unit time period considered. This term is proportional to the increase in the number of subscribers. In the telecommunication industry, this is given as a function of the a*verage revenue per user* (*ARPU*), with the scaling factor typically ranging from 4 to 8 [14]. The third and fourth terms combine to determine the cost of providing internet service to all subscribers of said service. We split this cost into two terms: the contractual cost, which is not dependent on the number of subscribers, and a subscription cost, which is proportional to the number of subscribers. The parameters in Eq (13) can be adjusted, some even set to 0 (e.g., growth rates), given the particular circumstances of an operator. We calculate the present value of the lifecycle costs as previously with Eq (3) and Eq (4), where the acquisition cost is given by Eq (12) and operational cost is given by Eq (13).

The revenue model of HTS is developed next, following which both (discounted) cost and revenue models are integrated to produce the *net present value* model of HTS.

### 3.2. Revenue model of HTS: Hybrid data-driven and scenario-based model

We break the development of the HTS revenue model into three parts for clarity: the load factor model, the subscribers-versus-throughput model, and finally, the service revenue model.

#### 3.2.1. Load factor model of HTS

**Fig 6:**
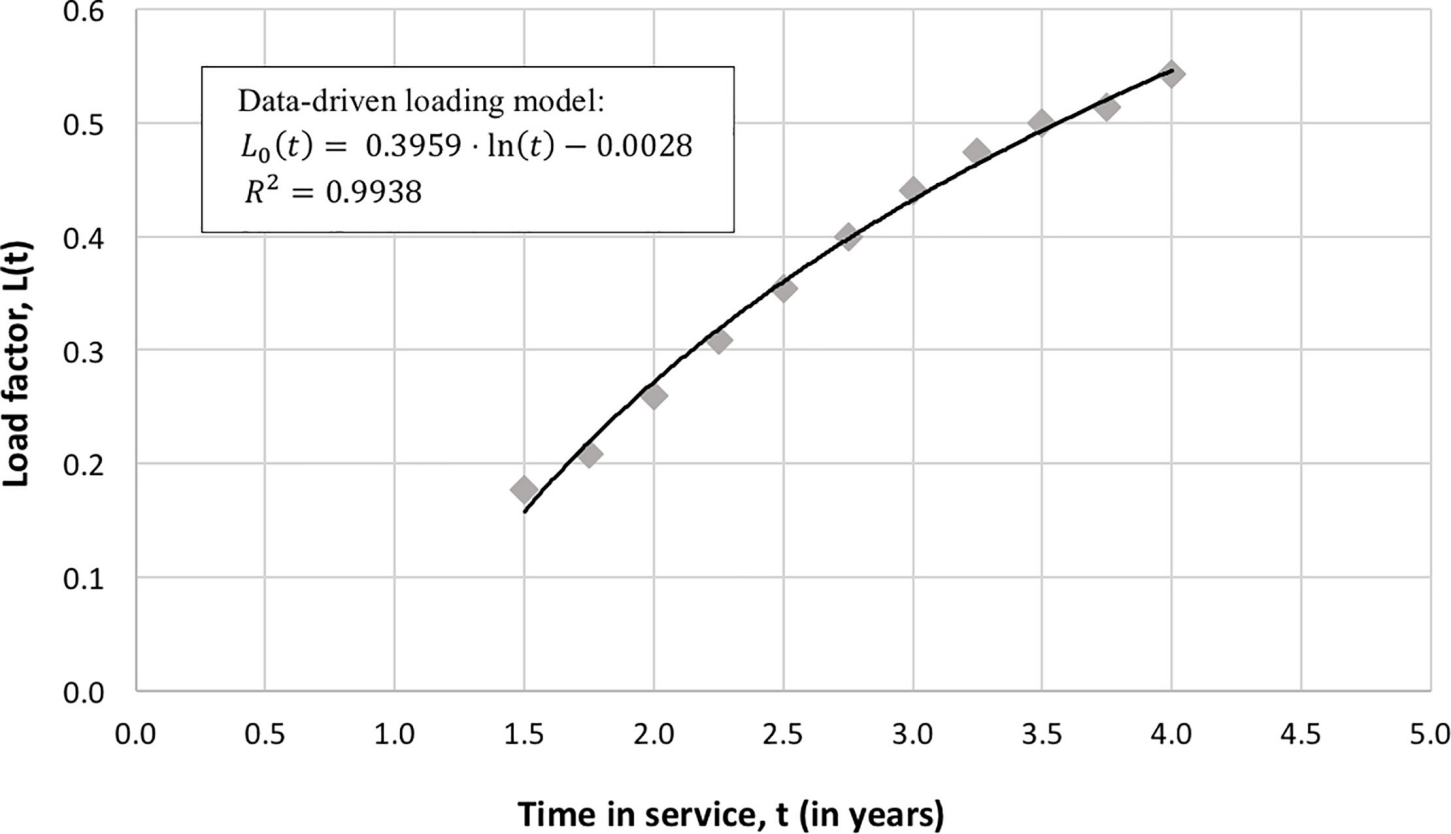
Loading data and regression model of KA-Sat load factor as a function of time.

As with wide-beams, a necessary component of the revenue model is the load factor. To construct the HTS load factor model, *L*_*HTS*_(*t*), we first examined market performance reports of KA-Sat, an HTS, launched in 2010 by Eutelsat [15, 16] and extracted its loading dynamics. Fig 6 provides a unique peak into an important but closely guarded piece of information by satellite operators. The best fit to this data was a logarithmic model, shown in Fig 6 and given by Eq (14) over the time interval for which the data was available.

$$\begin{array}{ccc} L_{HTS}(t)=0.3959\cdot \ln(t)-0.0028 & for & 1.5\leq t<4 \end{array}$$ (14)

**Fig 7:**
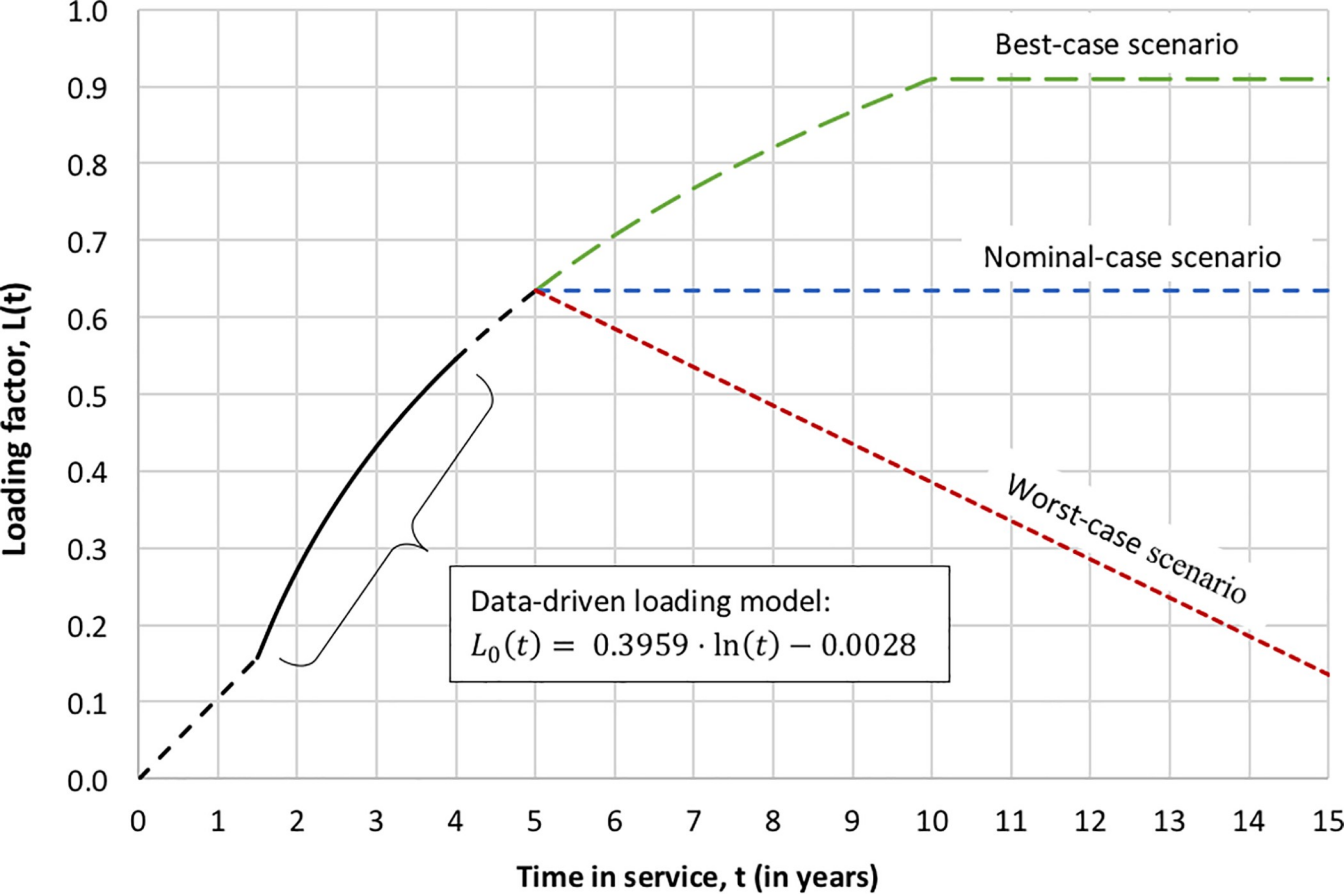
HTS loading profiles as a function of time with best-case, nominal-case, and worst-case scenarios.

The result shows a monotonic and slow (sub-linear) increase in the number of subscribers over the observational period. While this model is valuable, it is limited to the duration for which the data was available (and extractable). To extend this model over a typical 15-year design lifetime, we combine this empirical, data-driven loading model with forecasted scenarios described as best-case, nominal-case, and worst-case scenarios, as depicted in Fig 7 and discussed next. The three scenarios have a common loading profile up to the fifth year of operation; it includes a linear extrapolation for the first 1.5 years for which no data was available (this is a conservative assumption since satellite operators are likely to pre-sell some capacity before the asset becomes operational) and an extrapolation of the logarithmic model for an additional year beyond the data availability (4^th^ to 5^th^ year in service).

The first scenario is the best-case HTS loading model, *L*_*B*_(*t*). This model extends the logarithmic loading profile up to 10 years in service. It then reaches and maintains a steady-state load factor of 91% from *t* = 10 *to* 15 years, as shown in Fig 7 (subject to a noise factor with small variance, which accounts from some stochasticity in the loading dynamics). The piecewise best-case loading model scenario is given in Eq (15).

$$L_{B}(t)=\left\{ \begin{array}{ll} 0.1051\cdot t & for\;0\leq t<1.5\;yrs\\ 0.3959\cdot \ln(t)-0.0028 & for\;1.5\leq t<10\;yrs\\ 0.91 & for\;10\leq t\leq 15\;yrs \end{array}\right.$$ (15)

The second scenario is the nominal-case HTS loading model, *L*_*N*_(*t*). The logarithmic loading profile, in this case, reaches and maintains a steady-state load factor of 63% from *t* = 5 *to* 15 years, as shown in Fig 7. The piecewise nominal-case loading model scenario is given in Eq (16).

$$L_{N}(t)=\left\{ \begin{array}{ll} 0.1051\cdot t & for\;0\leq t<1.5\;yrs\\ 0.3959\cdot \ln(t)-0.0028 & for\;1.5\leq t<5\;yrs\\ 0.63 & for\;5\leq t<15\;yrs \end{array}\right.$$ (16)

Finally, the third scenario is the worst-case HTS loading model, *L*_*W*_(*t*). This loading profile includes the effects of obsolescence and customer churn. The logarithmic model extends till the 5^th^ year of service, as with the nominal scenario, but the load factor starts a linear decrease from *t* = 5 *to* 15 years, from a peak of 63% down to 13% by the end of the 15^th^ year of service (an average equivalent of 38% loading over this 10-year period). The piecewise worst-case loading model scenario is given in Eq (17).

$$L_{W}(t)=\left\{ \begin{array}{ll} 0.1051\cdot t & for\;0\leq t<1.5\;yrs\\ 0.3959\cdot \ln(t)-0.0028 & for\;1.5\leq t<5\;yrs\\ -0.05\cdot (t-5)+0.63 & for\;5\leq t<15\;yrs \end{array}\right.$$ (17)

The notation *L*_*HTS*_(*t*) will hereafter refer to one of these hybrid-loading models, depending on the scenario: best, nominal, or worst.

#### 3.2.2. Model of number of subscribers for HTS

Unlike the revenue model of traditional wide-beam communication satellites, with HTS, the number of subscribers is modeled as a function of the total throughput of the satellite, a system-level design parameter. The maximum number of subscribers, *N*_*max*_, is given by Eq (18).

$$N_{max}=\frac{R_{total}}{(S\cdot \eta_{1}\cdot \eta_{2})\cdot \mu }$$ (18)

$$\left\{ \begin{array}{ll} N_{max} & :maximum\;number\;of\;HTS\;subscribers\\ R_{total} & :total\;satellite\;throughput\\ S & :highest\;downlink\;speed\\ \eta_{1} & :ratio\;of\;average\;to\;highest\;downlink\;speeds\\ \eta_{2} & :ratio\;of\;actual\;to\;average\;downlink\;speeds\\ \mu & :average\;user\;activity\;(\%/user) \end{array}\right.$$

The product (*S*∙η_1_∙*η*_2_) denotes the actual average downlink speed. Multiplying this product with the average user activity per user, *μ*, we arrive at the actual average downlink speed per user. We define the maximum number of subscribers as the total throughput divided by the actual average downlink speed per user [17, 18].

#### 3.2.3. Model of service revenues for HTS

By combining the number of subscribers and the hybrid load factor models of HTS, we determine the average revenue generated per service for HTS per unit time period (quarter or year), ${\bar{u}}_{i,HTS}(t)$. This is given in Eq (19); it has been tailored for two modes of operation: when there is an increase in the number of subscribers and when there is a decrease. In the former case, additional revenue is generated when a new terminal to sold to a customer.

$${\bar{u}}_{i,HTS}(t)=\left\{ \begin{array}{lll} ARPU\cdot N_{max}\cdot L_{HTS}(t)+R_{t}\cdot \Delta N & for & \Delta N\geq 0\\ ARPU\cdot N_{max}\cdot L_{HTS}(t) & for & \Delta N<0 \end{array}\right.$$ (19)

The total revenue generated per unit time period for HTS, ${\bar{u}}_{total,HTS}(t)$, is given by Eq (20).

$${\bar{u}}_{total,HTS}(t)=\sum_{services}{\bar{u}}_{i,HTS}(t)$$ (20)

$$\left\{ \begin{array}{ll} ARPU & :average\;revenue\;per\;user\\ N_{max} & :maximum\;number\;of\;HTS\;subscribers\\ L_{HTS}(t) & :hybrid\;load\;factor\;model\;of\;HTS\\ R_{t} & :revenue\;generated\;by\;selling\;a\;transponder\\ \Delta N & :change\;in\;the\;number\;of\;subscribers \end{array}\right.$$

Note that the revenue does not decrease when the change in the number of subscribers, *ΔN*, is negative. The present value of HTS revenue is calculated as done previously via Eq (9), where the total revenue is given by Eq (20).

### 3.3. NPV model of high-throughput satellites

In parallel with Eq (10), we aggregate the present value of the revenue generated by HTS and the present value of the life cycle costs to obtain the *net present value* of HTS, *NPV*_*HTS*_, as given by Eq (21).

$$\begin{array}{c} NPV_{HTS}=PV(rev_{HTS})-PV(LCC_{HTS})\\ \Leftrightarrow \\ NPV_{HTS}=\sum_{i=1}^{T_{service}}\frac{{\bar{u}}_{total,HTS}(t)}{{\left(1+r\right)}^{i}}-\left[(C_{acq,HTS}+C_{launch})∙(1+IR)+\sum_{i=1}^{T_{service}}\frac{C_{ops,HTS}(t)}{{\left(1+r\right)}^{i}}\right] \end{array}$$ (21)

In the next section, we use this model to assess the value of HTS and determine primary drivers of the expected *NPV* and the associated uncertainty and tradeoffs.

## 4. Monte-Carlo value analysis of HTS: Results and discussion

In this section, we examine the *net present value* of HTS and identify key tradeoffs. First, we analyze the value of HTS under the three different loading scenarios discussed previously. We also provide a comparative analysis of the *NPV* of wide-beam satellites with that of HTS under the nominal-case loading scenario. Second, we identify the tradeoffs between *ARPU* and average loading for different times-to-break-even (and downlink speed). We also discuss the relevance and implications of each result.

The benchmark satellite was modeled as a medium-sized HTS with a design life of 15 years. We use a throughput capacity of 100 Gbps to highlight the throughput capacity threshold identified in Fig 5. We limit the random variables within the stochastic simulation environment to the launch cost and the *average revenue per user* (*ARPU*) to capture the dependence of these parameters on competitive intensity. The combination of remaining parameters was chosen to reflect the HTS that are likely to be in service in the near future. A full description of the parameters and random variables, along with their distributions, is provided in Table B in S1 Appendix.

### 4.1. HTS value analysis under different scenarios

**Fig 8:**
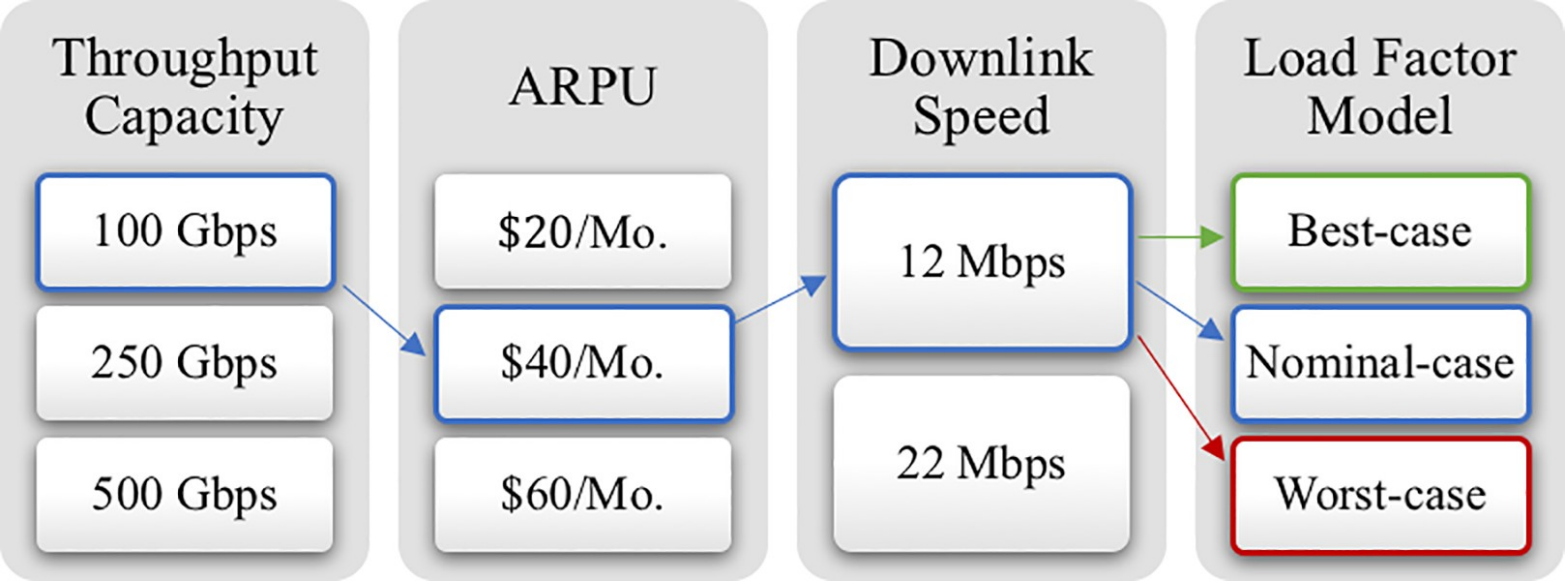
Discrete choices for some key HTS parameters. 54 different combinations were generated and value-analyzed. Results for the three highlighted scenario combinations are here presented.

Fig 8 displays four of the key value drivers and how they can be mixed and matched to generate different scenarios for the subsequent analysis. At the far left is the satellite throughput capacity, which is a system-level design choice. Next is the *ARPU*, which is a pricing decision, driven by operational considerations and competitive intensity within the market of interest. This is closely related to the next parameter, the downlink speed offered to consumers. This is both a technical as well as an operational and marketing decision. Finally, at the far right of Fig 8 is the *aggregate* load factor model (not at the level of a single consumer, unlike the *ARPU* and downlink speed), and it is here captured by the three loading scenarios discussed previously. The coupling between the last three parameters, *ARPU*, downlink speed, and loading factor, is beyond the scope of the present work. Nevertheless, this *ARPU* / downlink speed elasticity of demand is an important matter for satellites operators to explore.

We selected, for this subsection, discrete sets of choices for each of these four parameters to facilitate the analysis and interpretation. These are realistic values, e.g., 100, 250, 500 Gbps satellite capacity (medium, large, and very large HTS [19, 20]); $20, $40, $60/Mo *ARPU*, the lower range is a bit aggressive but plausible within this decade; and 12–24 Mbps downlink speed, the upper range is a bit aggressive but also plausible within this decade. Collectively, these discrete choices offer a reasonable picture of HTS design, market offerings, and performance. We examined the 54 different combinations that can be generated by mixing the different discrete choices of parameters. To keep this work within a manageable length, we only discuss the three scenarios highlighted in Fig 8.

**Fig 9:**
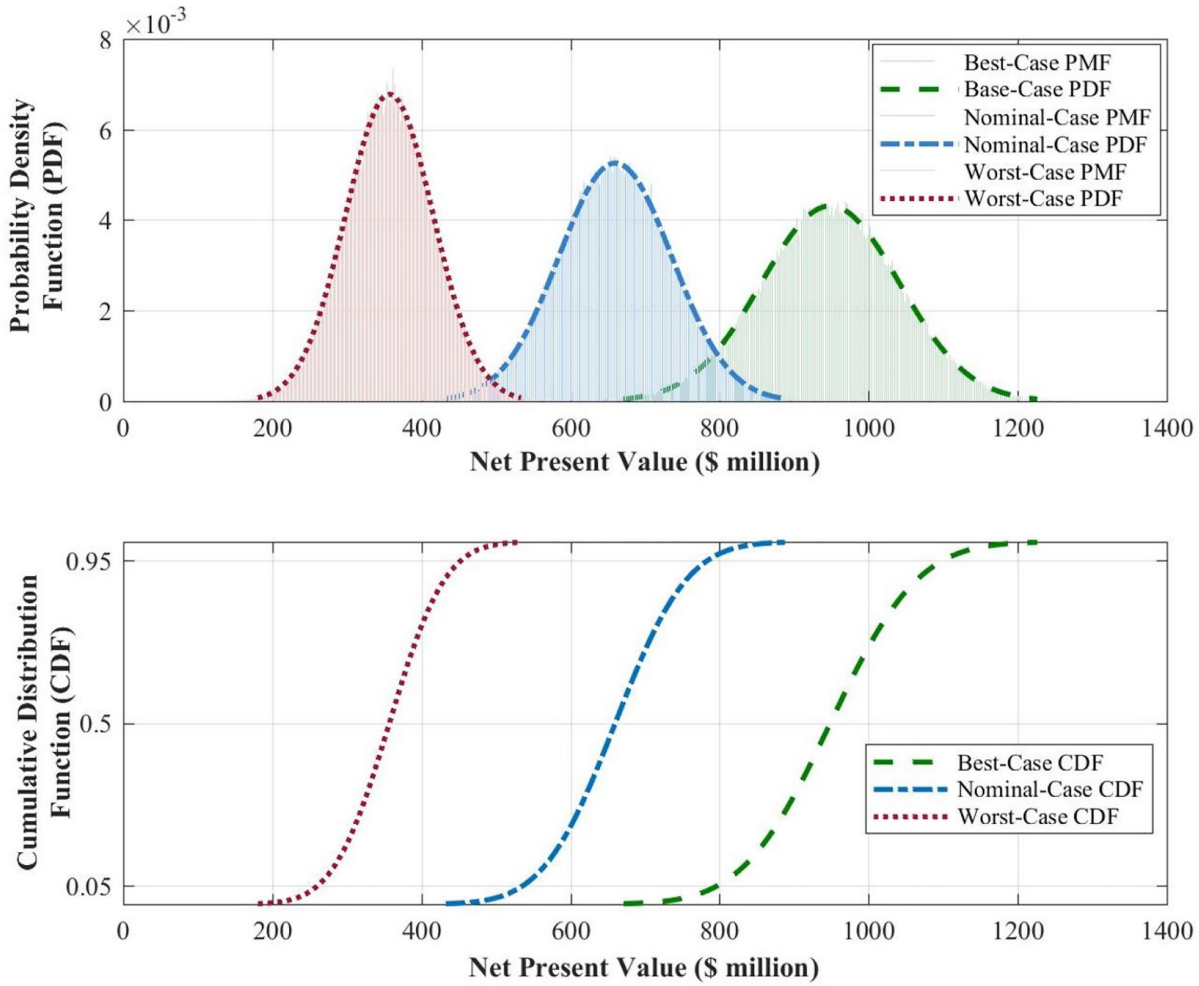
*NPV* analysis of HTS under three scenarios best-, nominal-, and worst-case loading conditions for a medium-sized HTS with a throughput capacity of 100 Gbps, *ARPU* of $40/month, and a downlink speed of 12 Mbps over a design life of 15 years on-orbit (details in Table B in S1 Appendix).

The results for these three scenarios are provided in Fig 9. They show, for example, that for the nominal-case scenario, the expected value of the *NPV* will be $660*m* with a standard deviation of $76*m* after 15 years on-orbit. Furthermore, the 90% confidence interval for this scenario spans the $535*m* to $785*m* range. The coefficient of variation for this representative satellite and market conditions is about 11.5%.

The salient results in Fig 9 are the following:

- Under all loading scenarios, this medium-sized HTS is *NPV*-positive, and it significantly outperforms the previous roughly equivalent (in terms of size/cost) wide-beam satellite;
- The load factor, as expected, is a significant driver of value, with the expected *NPV* increasing from $357*m* to $949*m* between the worst-case and best-case loading scenarios (see Fig 7 and the subsequent discussion for the loading scenarios details);
- The *NPV* volatility for HTS, whether reflected by the standard deviations, the 90% confidence intervals, or the coefficient of variations, remains manageable, with significant upside potential and no downside risk (likelihood of *NPV*<0). Its dimensionless measure is roughly similar to that of the previous wide-beam satellite.

**Fig 10:**
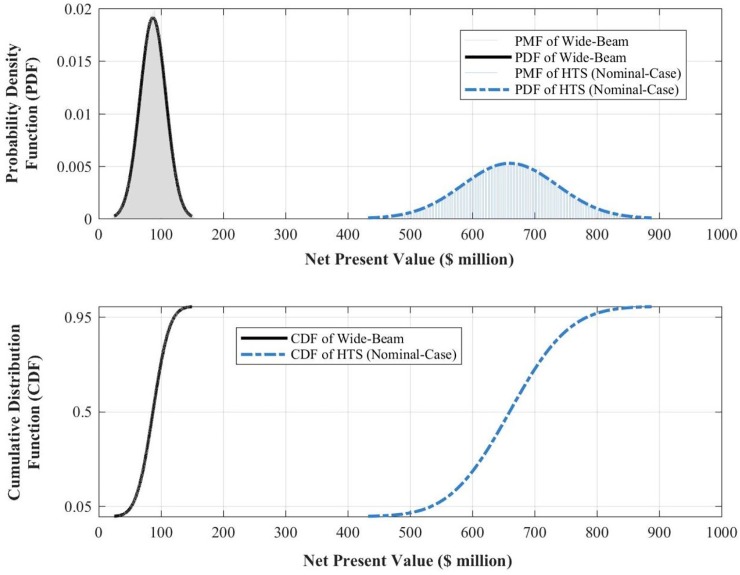
*NPV* distributions of the wide-beam satellite and the medium-sized HTS under the nominal-case scenario.

Fig 10 displays the PDF and CDF of both the previous wide-beam satellite and the medium-sized HTS considered in this work. Only the nominal scenario for the HTS is displayed to avoid visual clutter and underscore the salient result noted previously.

**Fig 11:**
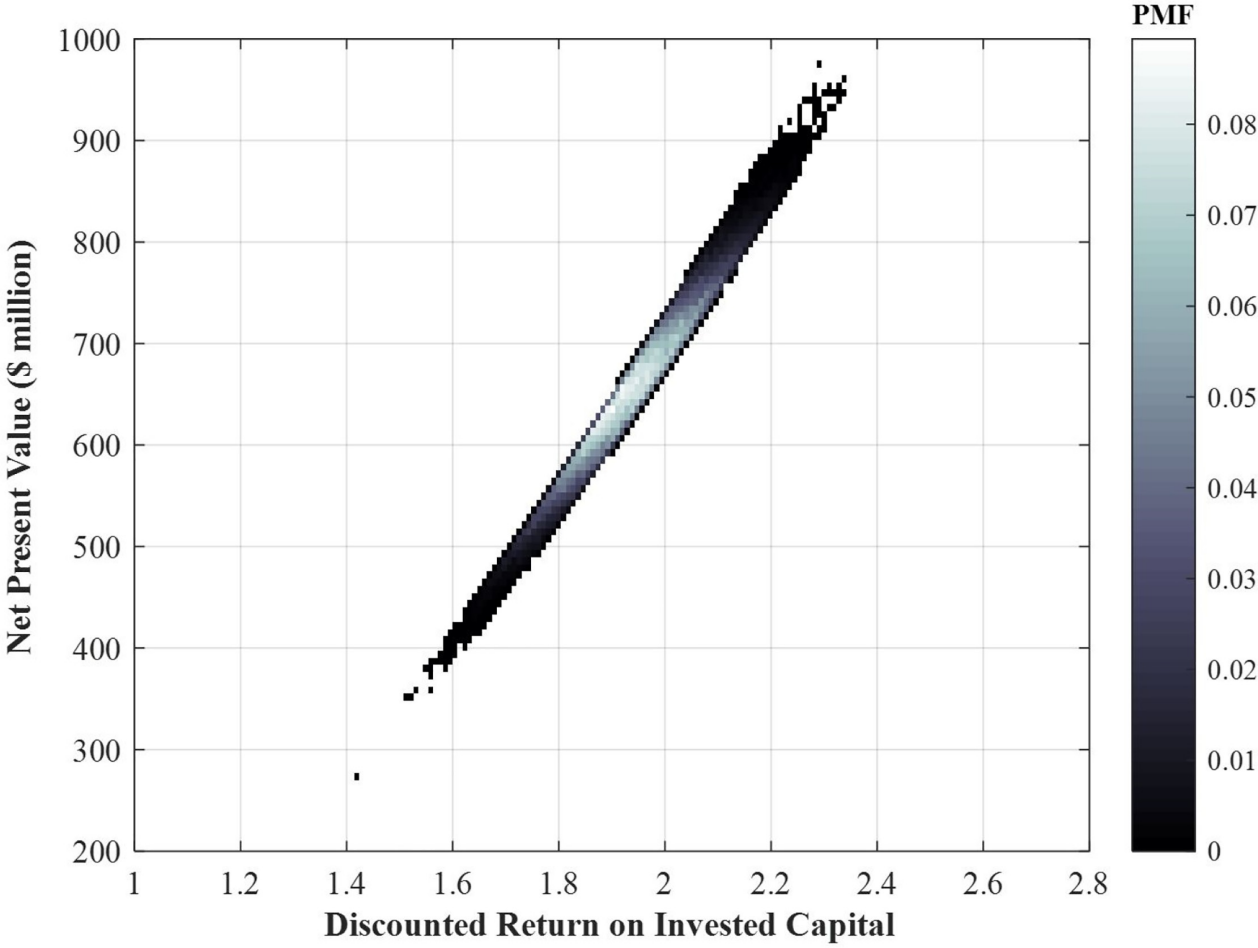
Joint distribution histogram of the *NPV* and *d-ROIC* for the medium-sized HTS under the nominal-case scenario after a design life of 15 years.

Fig 11 provides the joint distribution of this medium-sized HTS *NPV* and *d-ROIC*.

The joint distribution histogram of Fig 11 indicates a strong coupling between the *NPV* and *d-ROIC*, with an average slope of about $7.6*m*/%. For the HTS considered here, under the nominal-case scenario, the expected values of the *NPV* and *d-ROIC* are $660*m* and 1.9 or 90% returns respectively. Figs 9 and 11 reflect a significant (financial) margin for the medium-sized HTS under the realistic design, operational, and market conditions here considered.

### 4.2. Beyond the discrete scenarios: Critical tradeoffs in HTS

In this subsection, we relax the assumption of specific discrete scenarios adopted previously and examine the problem with a continuous range of *ARPU* and an average loading of the satellite, ${\bar{L}}_{0}$. More specifically, we set the objective of meeting a target *NPV* = 0 within a given time period, or time to break-even, and we analyze the contour plots (curves) within the $ARPU-{\bar{L}}_{0}$ space which achieve this objective. We maintain, nonetheless, a discrete set of downlink speeds for ease of interpretation and to avoid the more involved visualization of the contour plots in 3D.

**Fig 12:**
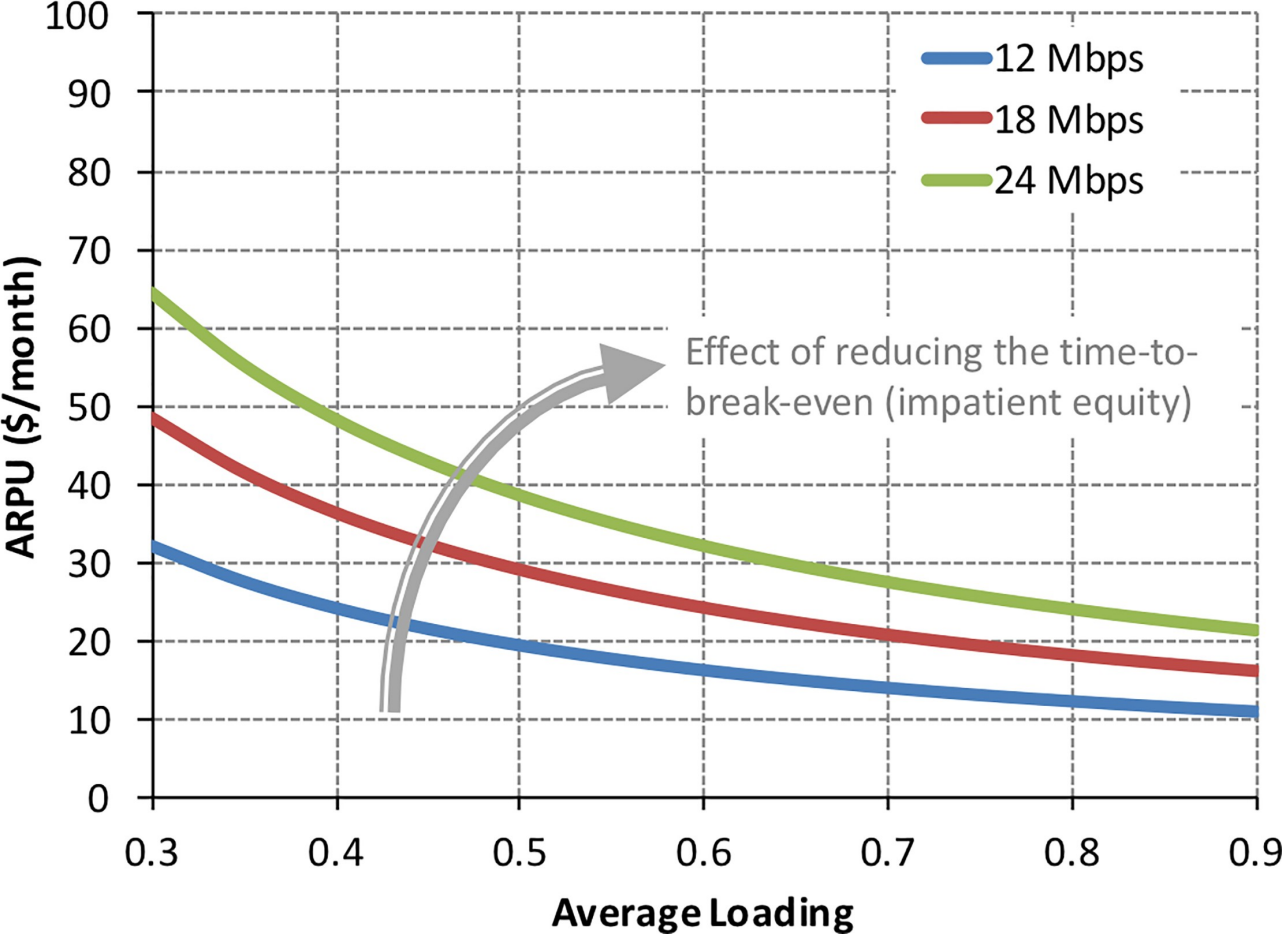
Required *ARPU* versus average loading for the medium-sized HTS to break-even in 10 years.

We chose this formulation because it helps identify and highlight several important tradeoffs for satellite operators to successfully manage their HTS. The results for a 10-year break-even horizon are provided in Fig 12.

Fig 12 shows the expected, and here quantified, tradeoff between *ARPU* and average loading of a medium-sized, 100 Gbps HTS. The figure reads as follows: consider, for example, a downlink speed of 18 Mbps and an *ARPU* of $30/month. An average loading of roughly 50% is required to break-even in 10 years. The required average loading increases to 70% if the *ARPU* drops to $20/month. Conversely, an *ARPU* of $50/month is required if an average loading of only 30% can be achieved. This is one reading grid of the results in Fig 12, and it constitutes an important input for an HTS operator to inform the pricing and marketing of its services.

A second reading grid for the results is the effect of increasing the downlink speed. One can read this result along a vertical or horizontal slice of Fig 12. Consider first a vertical slice: for an average loading of 50%, the *ARPU* required to break-even in 10 years is $30/month for a downlink speed of 18 Mbps. This *ARPU* increases to $40/month if a downlink speed of 24 Mbps is offered to customers. More generally, a higher *ARPU* is required to break-even if a higher downlink speed is provided to customers, all else being equal (average load factor). Conversely, a horizontal slice along Fig 12 indicates that if the *ARPU* is to be maintained (e.g., marketing insists that competitive intensity precludes any increases to *ARPU*), a higher downlink speed requires a higher average loading factor for the satellite to break-even in 10 years.

Fig 12 provides realistic boundaries for this HTS to break-even within the time horizon here considered. If an average loading factor of 40% to 60% can be achieved, the pricing of the service (*ARPU*) can be realistically set somewhere between roughly $20/month and $50/month. These are competitive price points in today’s terrestrial broadband delivery.

**Fig 13:**
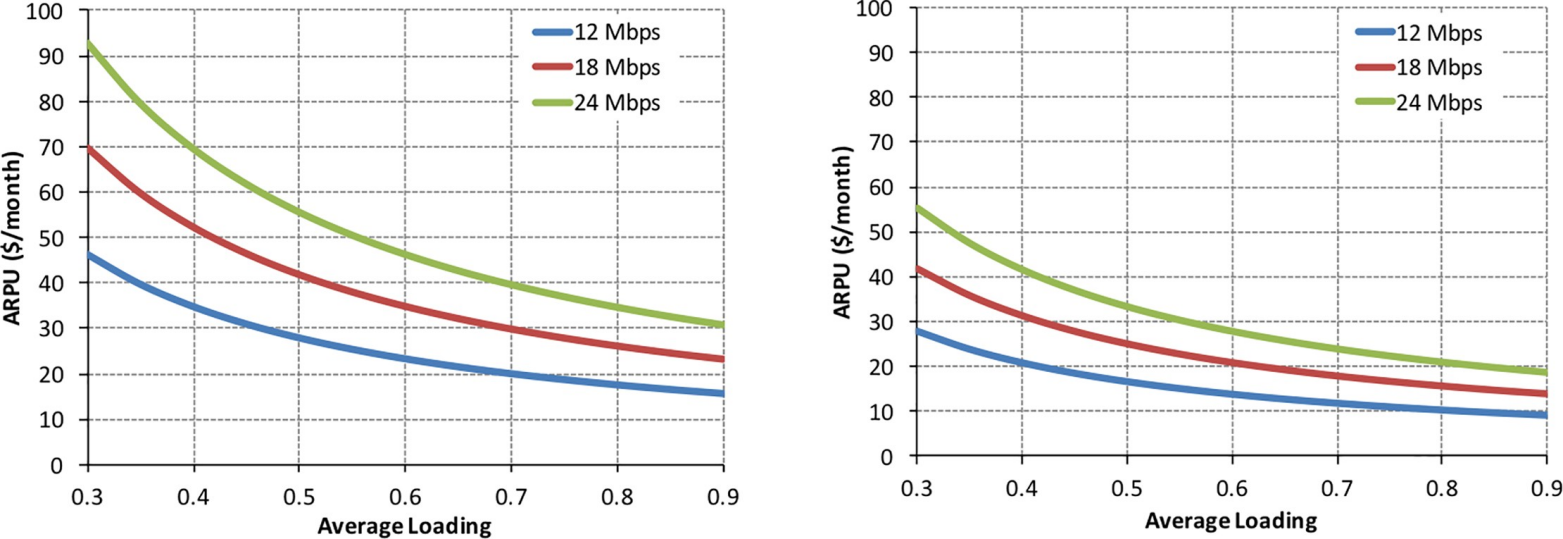
Required *ARPU* versus average loading for the medium-sized HTS to break-even in 5 years (left panel) and 15 years (right panel).

One last result, casually indicated in Fig 12, is the effect of changing the time to break-even on the tradeoff between *ARPU* and average load factor. The results were not included in Fig 12 to avoid excessive visual clutter. They are instead provided in Fig 13 for two discrete choices of time to break-even, namely 5 years and 15 years. Modifying the time to break-even reflects the “patience” of the equity investors or satellite operators have with respect their asset, namely the HTS. Different stakeholders can have different time horizons of interest. A hedge fund, for example, may be more interested in a 5-year time horizon, whereas a large corporation, such as Google or Amazon, has “patient equity” and can afford a longer time horizon of interest. The important point for our purposes is how this time horizon, here coarsely captured by the time to break-even, affects the tradeoff between *ARPU* and average load factor. Figs 12 and 13 show that if HTS operators can afford a longer time to break-even, they can, as expected, lower the *ARPU* for a given average loading. For example, at 50% loading, the *ARPU* required to break-even in 5 years is about $40/month, and it drops to $25/month when the time to break-even is 15 years—an extreme value (*ARPU* = $25/*month*), given the design lifetime of the satellite—but an important asymptotic value nonetheless. The results are increasingly more sensitive to the time to break-even at low average loading of the satellite.

## 5. Conclusion

The objective of this work was both to develop a decision-analytic framework for assessing the value of high-throughput satellites and to provide meaningful results of the value of such systems under realistic design, operational, and market conditions. These are meant to help inform design, cost, and service pricing considerations, among other things, for these systems to be successful.

In the process of developing these analytics, we derived an intermediate result, namely the power relationship between HTS affordability (cost per Gbps) and throughput, which reflects clear and substantial economies of scale in the cost of connectivity of these systems, with the “knee” in the curve occurring around 100 Gbps. The implication of this finding is that it will become increasingly more difficult to justify the design and acquisition of small GEO HTS below this throughput threshold.

More importantly, we found that a medium-sized HTS significantly outperforms a roughly equivalent traditional wide-beam satellite in terms of *NPV*, even under the worst-case loading scenario. The implication of this result is best conveyed by paraphrasing a poem by John Donne and used by Hemingway, “*therefore send not to know for who the bell tolls*, *it tolls for* [the wide-beam satellites].” Their obsolescence is forthcoming, even though their niche market may continue to be served by hybrid wide beam/spot beams satellites for a short while in the near future.

Another important result here identified and quantified is the tradeoff between *ARPU* and average loading of the satellite and how it is mediated by the downlink speed provided to consumers. This result can be used in different ways, for example, by helping define the boundaries of what is competitively achievable (in terms of *ARPU* and downlink speed offerings) and by adjusting accordingly the pressure on customer acquisition to reach a target loading factor. The implications of these results are that they clearly delineate the pathways to financial failure and the boundaries beyond which an HTS will not be value-positive (or alternatively, the asymptotic minimum values for an HTS to be value-positive).

Two important limitations and underlying assumptions of this work should be acknowledged. The first was noted in the previous section, and it concerns the elasticity of demand and satellite loading to changes in *ARPU* and downlink speed. In this work, these three parameters, average loading, *ARPU*, and downlink speed, were treated as independent. In reality, there will be coupling between these parameters, and the corresponding relationships will be functions of the competitive intensity for broadband delivery, or lack thereof, in a given market. What form these relationships might take (e.g., what types of non-linearities or discontinuities might ensue) and how to explore them, is left as a fruitful venue for future work. Nevertheless, it is important to recognize that the value of an HTS will be contingent to some extent on these *elasticities*, and satellite operators should prioritize understanding these relationships in the markets they seek to serve.

The second limitation is more surreptitious, and it concerns the way the value of the satellite was assessed. The underlying assumption in our calculations is that of the traditional broadband business model in which the *subscriber* is also the *payer*, and the aggregate *ARPU* across all subscribers constitutes the major revenue stream that feeds into the value calculations. This need not be the only business model and value streams of HTS broadband delivery. For example, in seeking to “connect the unconnected,” that is, the 4 billion individuals worldwide currently estimated to lack internet access [2], satellite operators may opt to provide broadband access for free in certain areas of the world, and other stakeholders (third parties) who can benefit from having access to data from a large consumer base for marketing, advertisement, or other data-related services would foot the bill (e.g., Google products for consumers, or Facebook). This new business model would be particularly well suited for corporations with both deep pockets and patient equity, and those that are both vertically integrated and already providing advertising and consumer product services. If such a business model is adopted, the new HTS revenue streams ought to be accounted for in the value analysis. The present work will need to be modified accordingly.

Finally, we note that the analyses and results in this article were confined to a single GEO HTS. How the decision-analytic framework here developed can be adapted to LEO HTS constellations will be explored in a follow-up work.

## Supporting information

S1 Appendix Value model parameters for Monte-Carlo value analyses. (DOCX)
