# Supplementary material for: Monte-Carlo value analysis of High-Throughput Satellites: Value levers, tradeoffs, and implications for operators and investors
Source: PLoS One. 2019 Sep 11;14(9):e0222133. doi: 10.1371/journal.pone.0222133 (PMC6738660; doi:10.1371/journal.pone.0222133)
Supplement: S1 Appendix — (DOCX) [file pone.0222133.s001.docx]

**S1 Appendix**

**Table A. Cost and revenue parameters of the value model for our traditional wide-beam communication satellite subject to Monte-Carlo stochastic value analysis.**

| Parameter | Distribution | Numeric Value(s) |
| --- | --- | --- |
| Number of simulations | N/A | 100,000 |
| Number of transponders | N/A | 60 |
| Design Lifetime [Yrs.] | N/A | 15 |
| Delta T [Yrs.] | N/A | 2 |
| Initial operation cost [$ millions] | N/A | 10 |
| Discount rate [%] | N/A | 10 |
| Estimated service year [Yrs.] | N/A | 15 |
| Steady-state load [%] | N/A | 80 |
| Load factor [%] | N/A | 0.5 |
| Marginal cost of durability [%] | Normal | μ = 3, σ = 0.5 |
| Launch cost [$ million] | Uniform | 80-120 |
| Insurance rate [%] | Normal | μ = 0.15, σ = 0.025 |
| Annual cost growth rate of operation cost [%] | Normal | μ = 3, σ = 0.5 |
| Annual cost growth rate of operation cost [%] | Normal | μ = 3, σ = 0.5 |
| Onset obsolescence [Yrs.] | Normal | μ = 9, σ = 1 |
| Intensity obsolescence [Yrs.] | Normal | μ = 20, σ = 2 |
| Fraction of video service [%] | Normal | μ = 35, σ = 5 |
| Fraction of audio service [%] | Normal | μ = 35, σ = 5 |
| Price of video service per transponder per year [$ million] | Normal | μ = 1.5, σ = 0.1 |
| Price of audio service per transponder per year [$ million] | Normal | μ = 1.4, σ = 0.1 |
| Price of data service per transponder per year [$ million] | Normal | μ = 1.6, σ = 0.1 |

**Table B. Cost and revenue parameters of the value model of our high-throughput satellite subject to Monte-Carlo stochastic value analysis.**

| Parameter | Distribution | Numeric Value(s) |
| --- | --- | --- |
| Number of simulations | N/A | 100,000 |
| Throughput capacity [Gbps] | N/A | 100 |
| Design lifetime [years] | N/A | 15 |
| Discount rate [% per year] | N/A | 10 |
| Terminal cost [$ million] | N/A | 300 |
| Initial annual operational cost [$ million] | N/A | 10 |
| Annual operational growth rate [% per year] | N/A | 0 |
| Customer acquisition cost ARPU factor [integer] | N/A | 8 |
| IPS initial annual cost [$ million per year] | N/A | 10 |
| IPS annual growth rate [% per year] | N/A | 0 |
| Quarterly percentage factor of ARPU [% per quarter] | N/A | 5 |
| Annual insurance rate [% per year] | N/A | 12 |
| Launch cost [$ million] | Uniform | 60-100 |
| Average revenue per user [$ million per month] | Normal | $\mu=40$*,* $\sigma=10$ |
